# Adding low dose cyclophosphamide to rituximab for remission-induction may prolong relapse-free survival in patients with ANCA vasculitis: A retrospective study

**DOI:** 10.1016/j.jtauto.2022.100178

**Published:** 2022-12-15

**Authors:** Renée Ysermans, Matthias H. Busch, Joop P. Aendekerk, Jan G.M.C. Damoiseaux, Pieter van Paassen

**Affiliations:** a Department of Internal Medicine, Division of Nephrology and Clinical Immunology, Maastricht University Medical Center, P. Debyelaan 25, 6202AZ, Maastricht, the Netherlands; b Central Diagnostic Laboratory, Maastricht University Medical Center, P. Debyelaan 25, 6202AZ, Maastricht, the Netherlands

**Keywords:** ANCA, Vasculitis, Rituximab, Cyclophosphamide, Relapse, Outcome

## Abstract

**Objective:**

Rituximab (RTX) and cyclophosphamide (CYC) are effective remission-induction therapies in anti-neutrophil cytoplasmic antibody (ANCA)-associated vasculitis (AAV). However, combining these therapies may favor prognosis in patients with a major disease presentation. We conducted a retrospective study to compare patients treated with a combination of RTX and low dose CYC (RTX-CYC) or with RTX only, both followed by tailored maintenance with RTX, with regard to long-term outcomes.

**Methods:**

Patients treated in the Maastricht University Medical Center between March 2007 and January 2019, were screened for eligibility. The primary outcome variable was major relapse rate after two and five years. Secondary outcome variables were clinical data and laboratory parameters.

**Results:**

Of the 246 screened patients, 34 received RTX-CYC and 28 RTX only for remission-induction. All patients were followed for at least two years, with a median follow-up of 48 months (IQR 24–60). At baseline, renal involvement was more prevalent in the RTX-CYC patients (85% vs. 61%, *P* = 0.028). Major relapse rates within two years, but not after five years, were significantly lower in the RTX-CYC group (3% vs. 24%, *P* = 0.032). The rate of infections, hypogammaglobulinemia, end-stage renal disease, malignancies, and mortality did not differ after two and five years.

**Conclusion:**

Adding low dose CYC to RTX is safe and may prevent major relapses in patients with severe AAV in the first two years after remission-induction. Randomized controlled trials that compare the efficacy and safety between RTX and a combination of RTX with CYC are needed.

## Highlights

• The addition of low-dose CYC to RTX for remission-induction prolongs relapse-free survival in AAV.
• There is no difference in adverse events between RTX-CYC and RTX for remission-induction in AAV.

## 1 Introduction

Anti-neutrophil cytoplasmic antibody (ANCA)-associated vasculitis (AAV) consists of granulomatosis with polyangiitis (GPA), microscopic polyangiitis (MPA), and eosinophilic granulomatosis with polyangiitis (EGPA). This group of small-to medium-sized vessel vasculitides is characterized by antibodies against myeloperoxidase (MPO) and proteinase-3 (PR3) in most patients and can result into life-threatening end-organ damage [1,2].

Remission-induction with cyclophosphamide (CYC) and rituximab (RTX), either separately or combined, in combination with glucocorticosteroids (GCS), have proven to be effective in patients with AAV [[3], [4], [5], [6]]. After achieving remission, azathioprine (AZA) or RTX are currently recommended as maintenance therapy for at least two years [[7], [8], [9], [10]]. There are no differences between patients treated with either RTX or a combination of RTX and low dose CYC and patients treated with CYC with regard to sustained remission and adverse events within two years [4,5,11,12]. Comparative data on the efficacy and safety of RTX monotherapy compared with RTX combined with low dose CYC, however, have not yet been published, but the major relapse risk appears to be lower when CYC is added to RTX [11,12]. This might point towards an advantage of RTX combined with CYC over RTX alone, especially in patients with severe organ involvement, such as a rapidly progressive glomerulonephritis, to prevent further tissue damage in the future.

In this study, we retrospectively assessed the frequency of major relapses and the safety profile (i.e., infections, end stage renal disease, malignancy, and mortality) at two and five years after remission-induction with either RTX combined with low dose CYC or RTX alone, followed by a tailored maintenance with RTX, in patients with AAV. We did not include patients treated with fixed maintenance with RTX, to keep the maintenance phase as homogenous as possible between the two different remission-induction regimens.

## 2 Methods

### 2.1 Study population

This is a retrospective single-center cohort study of patients with AAV that were treated with a RTX based remission-induction scheme between March 2007 and January 2019 followed by a patient tailored RTX maintenance regimen at the Maastricht University Medical Center. The diagnosis of AAV was made according to the revised Chapel Hill criteria [1]. Patients with a new diagnosis of AAV or relapsing disease who were followed for at least two years were included. Inclusion started at remission-induction therapy and continued until death, loss to follow-up, or the latest update in January 2021.

The study was performed in accordance with the 1997 Declaration of Helsinki and ethical approval from the local Institutional Review Board was obtained (2020–2359). The data underlying this article will be shared on reasonable request to the corresponding author.

### 2.2 Treatment

For remission-induction therapy, patients were treated with RTX (two doses of 1000 mg within two weeks) combined with CYC (two intravenous doses of 15 mg per kilogram within two weeks or an oral equivalent), or with RTX alone (two doses of 1000 mg within two weeks). The first treatment group will be referred to as RTX-CYC and the second treatment group as RTX only. Additionally, all patients received methylprednisolone (three intravenous doses of 500–1000 mg per day) or prednisolone (1 mg/kg orally with a maximum of 60 mg per day) with a subsequent individualized tapering scheme. The decision of which remission-induction scheme to use was not randomized, but in our tertiary expertise center CYC based schemes tend to be more frequently prescribed to patients with rapidly progressive or severe renal involvement (i.e. CKD4 or 5).

Maintenance therapy with RTX (one dose of 1000 mg) was patient tailored and given in case of CD19^+^ B cell repopulation (≥5 cells/μL), combined with a meaningful rise in ANCA level (i.e. doubling) and/or clinical symptoms, without meeting the criteria of a major relapse.

### 2.3 Data collection

The data were collected by reviewing the patient records independently by the authors. The patient characteristics age, sex, AAV type, ANCA specificity, date of diagnosis and relapse, organ involvement, Birmingham Vasculitis Activity score (BVAS) [13] at the time of a new diagnosis or relapse, remission-induction and maintenance regimens, time to complete remission and time to relapse, complications before and after treatment, mortality, as well as biochemical data, were collected. Whenever the BVAS before remission-induction was not noted in the patient's record, the BVAS was scored by two trained physicians based on the available data. When kidney biopsies were performed, these were described using Berden's histopathologic classification for glomerulonephritis in AAV [14]. In addition, ANCA renal risk scores were calculated, which is a validated method for the prediction of end stage renal disease (ESRD) by combining histopathologic features and renal function during disease activity [15].

### 2.4 Definitions

Remission was defined by a Birmingham Vasculitis Activity Score (BVAS) of 0. Major relapse was defined as the recurrence of clinical symptoms associated with a major BVAS item, indicating major organ involvement, requiring remission-induction therapy [13].

### 2.5 Outcome variables

The primary outcome variable was major relapse rate after two and five years. Secondary outcome variables were clinical data including infections requiring treatment, ESRD, malignancy, and mortality and laboratory parameters including renal function, MPO- or PR3-ANCA and immunoglobulin levels, and circulating CD19^+^ B cells.

MPO- and PR3-ANCA levels were measured with ImmunoCAP using Phadia 250 (Thermo Fisher Scientific, Waltham, MA) [16]. The number of CD19^+^ B cells was quantified by flowcytometry [17].

### 2.6 Statistical analyses

Continuous variables were reported as median (interquartile range [IQR]), based on the distribution of the data. Baseline characteristics of the two treatment groups were compared with the non-parametric Mann-Whitney *U* test in case of numerical dependent variables and the Chi-square test or Fisher's exact test in case of categorical dependent variables.

Remission and relapse rates, as well as the occurrence of adverse events at two and five years, were analyzed with the log-rank/Mantel-Cox test and reported as hazard ratio's (HR) with 95% confidential intervals (CI). Patients who died or were not followed for five years, were marked as censored observations for the available follow-up period. Statistic analyses were performed with IBM SPSS Statistics (version 28) and GraphPad Prism (version 5). Statistical significance was assumed for a P-value <0.05.

## 3 Results

### 3.1 Study population

**Fig. 1:**
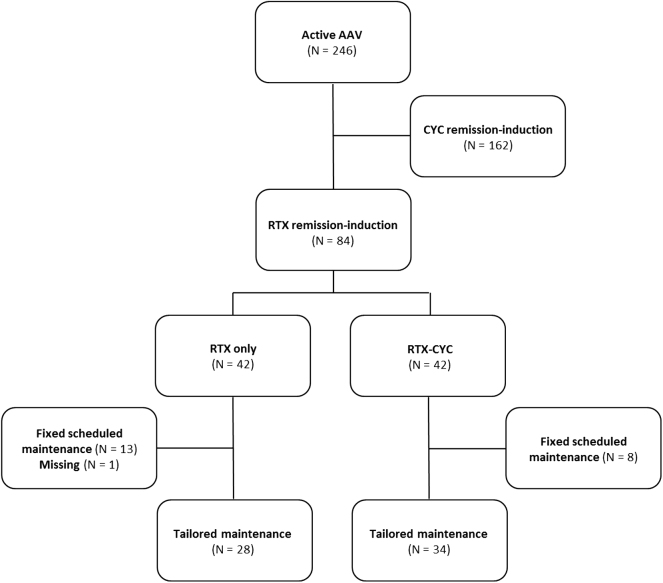
Flowchart of included patients. Abbreviations: AAV = anti-neutrophil cytoplasmic antibody-associated vasculitis, RTX = rituximab, CYC = cyclophosphamide.

Of the 246 patients with active AAV screened for the study, a RTX based remission-induction scheme with a subsequent tailored RTX maintenance regimen was given in 34 patients in the RTX-CYC and 28 patients in the RTX only group (Fig. 1). The baseline characteristics of the included patients are depicted in Table 1. Both groups were statistically similar at baseline in terms of sex, age, de novo presentation/relapsing disease, and disease activity, as well as biochemical parameters, except for renal involvement, which was more prevalent in the RTX-CYC group as compared to the RTX only group (85% vs. 61%, *P* = 0.028). Additional treatment with plasma exchange (PLEX) was given to seven patients treated with RTX-CYC with severe renal disease and/or alveolar haemorrhage and to none of the patients in the RTX only group (*P* = 0.016). Patients were regarded as having severe renal disease when the disease was rapidly progressive and/or in case of extensive activity in the kidney biopsy.

**Table 1:** Patient characteristics at baseline.

	RTX-CYC (N = 34)	RTX only (N = 28)	P-value^a^
**Male, n (%)**	17 (50)	19 (67.9)	0.156
**Age (years), median (IQR)**	63 (48–70)	60 (55–69)	0.896
**De novo presentation, n (%)**	12 (35.3)	9 (32.1)	0.794
**Disease duration (years), median (IQR)**	9 (3–13)	9 (4–17)	0.608
**ANCA, n (%)**			0.493
MPO	12 (35.3)	12 (42.9)	
PR3	21 (61.8)	14 (50.0)	
Double positive	0	1 (3.6)	
Other ANCA specificities^b^	0	1 (3.6)	
Negative	1 (2.9)	0	
**Diagnosis, n (%)**			1.000
MPA	8 (23.5)	7 (25.0)	
GPA	24 (70.6)	20 (71.4)	
EGPA	2 (5.9)	1 (3.6)	
**BVAS, median (IQR)**	14 (11–18)	12 (7–17)	0.164
**Organ involvement, n (%)**			
General	13 (38.2)	13 (46.4)	0.608
Cutaneous	4 (11.8)	4 (14.3)	1.000
Mucous membranes	4 (11.8)	6 (21.4)	0.326
ENT	20 (58.8)	16 (57.1)	1.000
Chest	20 (58.8)	15 (63.6)	0.798
Cardiovascular	2 (5.9)	2 (7.1)	1.000
Abdominal	3 (8.8)	0	0.245
Renal	29 (85.3)	17 (60.7)	**0.028**
Nervous system	4 (11.8)	5 (17.9)	0.719
**Laboratory data, median (IQR)**			
CRP (mg/L)	13.5 (4.3–66.0)	13.0 (2.0–65.0)	0.879
IgG (g/L)	9.0 (5.3–12.0)	10.5 (8.3–15.2)	0.251
**Renal parameters**^c^			
Serum creatinine (μmol/L), median (IQR)	126.0 (91.0–238.0)	164.0 (96.0–192.0)	0.499
Estimated glomerular filtration rate (ml/min/1,73m^2^), median (IQR)	44.5 (20.0–67.8)	40.0 (30.0–75.0)	0.284
Estimated glomerular filtration rate ≤30, n (%)	11 (37.9)	3 (17.6)	0.308
**Kidney biopsy, n (% of patients with renal involvement)**	24 (82.8)	11 (64.7)	0.282
**Berden classification kidney biopsy, n (%)**			0.791
Not active	1 (4.2)	0	
Focal	9 (37.5)	6 (54.5)	
Crescentic	1 (4.2)	1 (9.1)	
Mixed	5 (20.8)	2 (18.2)	
Sclerotic	8 (33.3)	2 (18.2)	
**ANCA renal risk score, n (%)**			1.000
Low risk	14 (58.3)	7 (63.6)	
Moderate risk	7 (29.2)	3 (27.3)	
High risk	3 (12.5)	1 (9.1)	

Abbreviations: RTX = rituximab, CYC = cyclophosphamide, ANCA = anti-neutrophil cytoplasmic antibody, MPO = myeloperoxidase, PR3 = proteinase 3, MPA = microscopic polyangiitis, GPA = granulomatosis with polyangiitis, EGPA = eosinophilic granulomatosis with polyangiitis, BVAS = Birmingham vasculitis activity score, ENT = ear, nose, and throat, CRP = C-reactive protein, IgG = immunoglobulin G.

a A p-value of 0.05 was considered significant.

b Azurocidin ANCA.

c In patients with renal involvement.

Within the two years after remission-induction, there were 14 (41%) patients with RTX-CYC who received a median of 1 (IQR, 1–2) maintenance infusion with RTX, with a median time to first administration of 10 (IQR, 6–12) months and 12 (43%) patients treated with RTX only who received a median of 1 (IQR, 1–2) RTX maintenance infusion, with a median time to first administration of 9.5 (IQR, 8–14) months (*P* = 0.755).

### 3.2 Remission and relapse rate

**Fig. 2:**
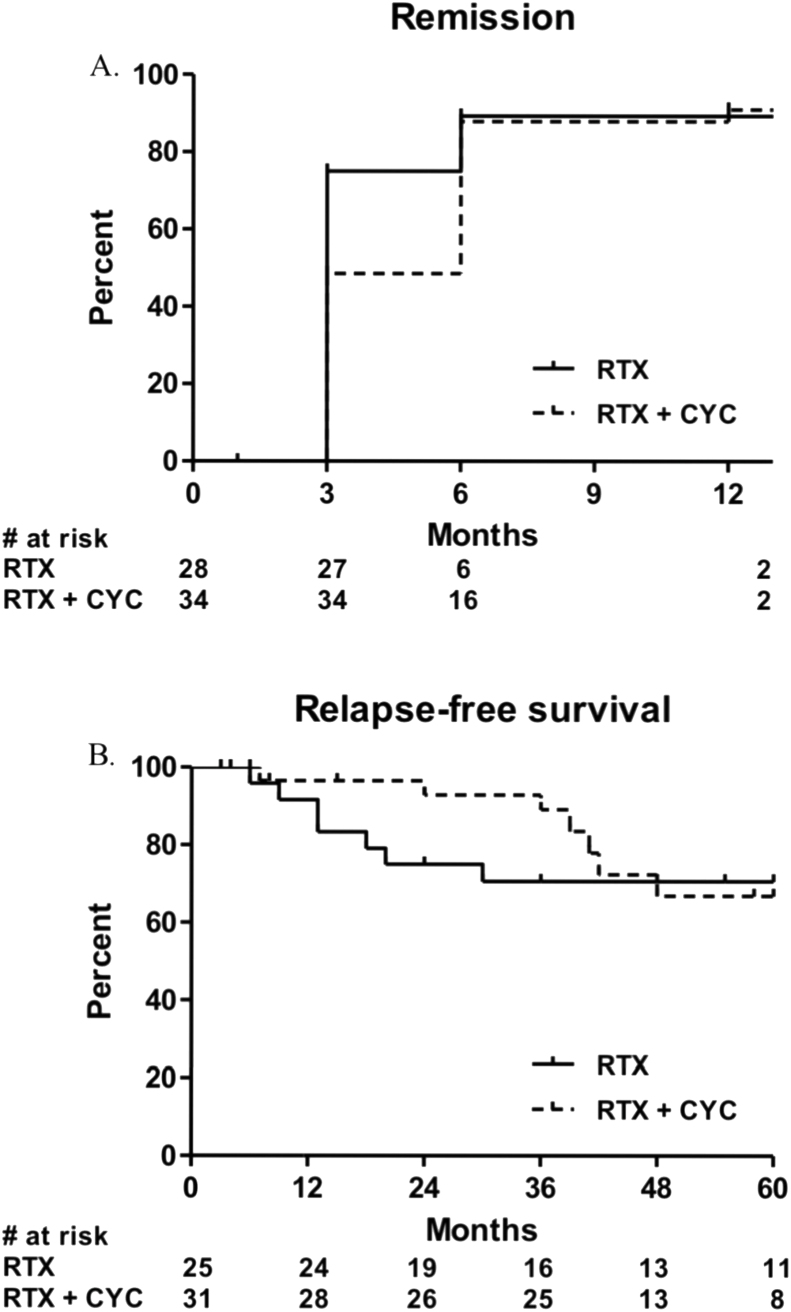
Cumulative percentage of patients in remission (A) and with relapse-free survival (B). Abbreviations: RTX = rituximab, CYC = cyclophosphamide. + = event/censored.

In the RTX-CYC group, 17 (50%) patients were in remission three months, 30 (88%) patients six months, and 31 (91%) patients twelve months after inclusion (Fig. 2A). In the RTX only group, 21 (75%) patients were in remission three months and 25 patients (89%) six as well as twelve months after inclusion (Fig. 2A).

Median tapering time to 5 mg per day prednisolone was 132 days (IQR, 92–180) in the RTX-CYC group versus 112 days (IQR, 75–175) in the RTX only group (*P* = 0.330).

Relapse rates were reported for patients who achieved remission (Fig. 2B). The relapse risk was significantly lower in patients treated with RTX-CYC compared to RTX only (n = 1 [3%] vs. n = 6 [24%]; HR 0.19 [95% CI 0.04–0.87], *P* = 0.032) within two years after remission-induction. A total of fourteen (n = 7 RTX-CYC vs. n = 7 RTX only) relapses were reported within five years of follow-up (HR 1.26 [95% CI 0.44–3.63], *P* = 0.666). Six (86%) patients in the RTX-CYC group had renal involvement at time of inclusion and three of these patients (43%) had a renal relapse. Four patients (57%) were newly diagnosed with AAV at time of inclusion. In the RTX only group, two (29%) of these patients had renal involvement at time of inclusion and one (14%) patient had a renal relapse. Three (43%) patients were newly diagnosed with AAV at inclusion.

### 3.3 Infections

**Fig. 3:**
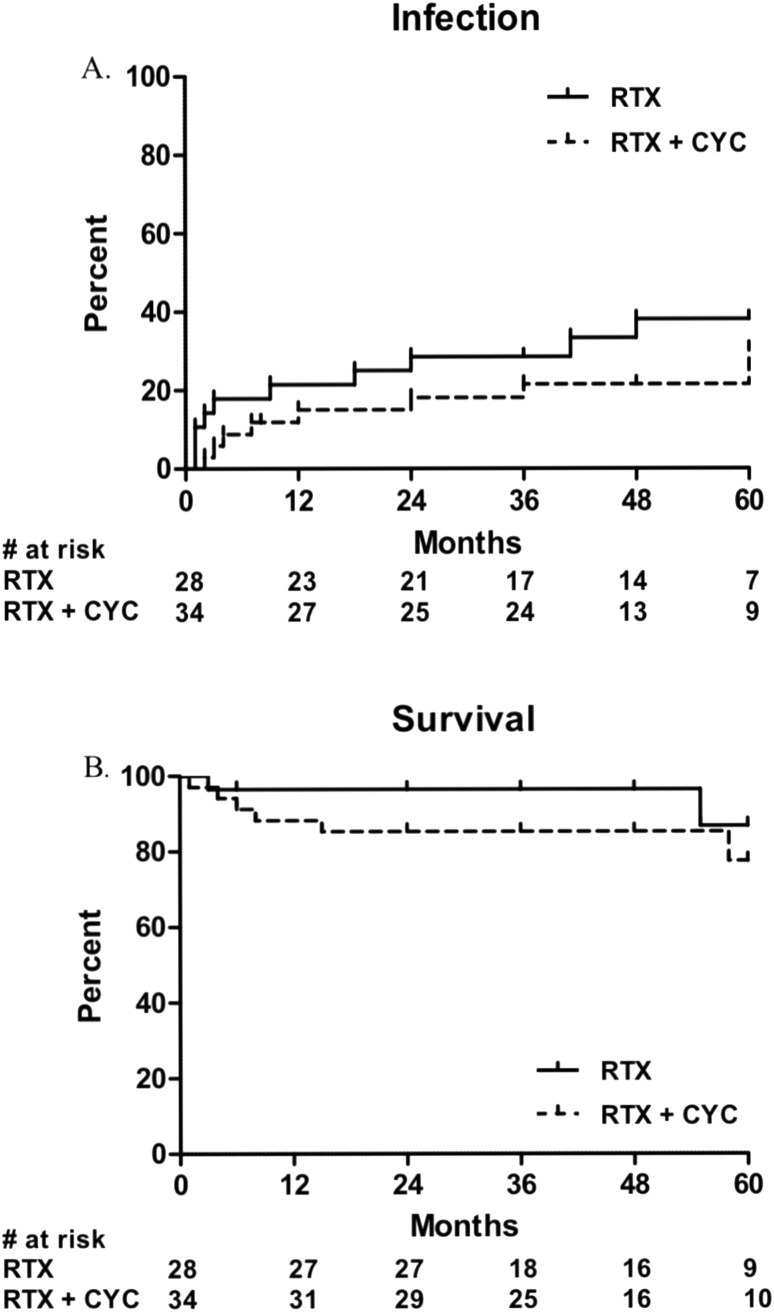
Cumulative percentage of patients with infections (A) and of patient survival (B). Abbreviations: RTX = rituximab, CYC = cyclophosphamide. + = event/censored.

Eight infections were observed in six (18%) patients treated with RTX-CYC vs. eleven infections in eight (29%) patients in the RTX only group (HR [0.58 (95% CI 0.20–1.69], *P* = 0.281) during two years of follow-up (Fig. 3A, Table 2). Three infections were fatal (n = 2 RTX-CYC vs. n = 1 RTX only). The number of infections further increased during five years of follow-up in boths groups (Fig. 3A, Table 3), but without statistically significant differences (RTX-CYC vs. RTX only, HR 0.64 [95% CI 0.25–1.62], *P* = 0.343). Infection types per treatment group are depicted in **Appendix** Table A1.

**Table 2:** Adverse events with hazard ratio's during the two year follow-up.

	RTX-CYC (N = 34)	RTX only (N = 28)	Hazard ratio (CI 95%) of adding CYC to RTX vs. RTX alone	P-value^a^
**Infections, n/number of patients (%)**	8/6 (17.6)	11/8 (28.6)	0.58 (0.20–1.69)	0.281
Requiring treatment	4	6		
Hospitalization	2	4		
Life threatening	0	0		
Death	2	1		
**Hypogammaglobulinemia, n (%)**	21 (61.8)	11 (39.3)	2.50 (0.95–6.54)	0.066
**ESRD, n (%)**	2 (5.9)	1 (3.7)	1.60 (0.16–15.81)	0.686
**Malignancy, n (%)**	1 (2.9)	2 (7.1)	0.43 (0.04–4.32)	0.585
**Death, n (%)**	5 (14.7)	1 (3.7)	3.25 (0.65–16.21)	0.214

Abbreviations: RTX = rituximab, CYC = cyclophosphamide, ESRD = end stage renal disease.

a A P-value of 0.05 was considered significant.

**Table 3:** Adverse events with hazard ratio's during the five year follow-up.

	Hazard ratio (CI 95%) of adding CYC to RTX vs. RTX alone	P-value^a^
Infections	0.64 (0.25–1.62)	0.343
Hypogammaglobulinemia	1.95 (0.86–4.45)	0.111
ESRD	2.20 (0.30–15.97)	0.436
Malignancy	0.43 (0.04–4.23)	0.473
Death	2.29 (0.57–9.22)	0.243

Abbreviations: RTX = rituximab, CYC = cyclophosphamide, CI = confidence interval, ESR D = end stage renal disease.

a A P-value of 0.05 was considered significant.

### 3.4 Hypogammaglobulinemia and intravenous immunoglobulin

Twenty-one (62%) patients in the RTX-CYC group and eleven (39%) patients in the RTX only group had hypogammaglobulinemia (IgG <7.0 g/L) within two years after remission-induction therapy (HR 2.50 [95% CI 0.95–6.54], *P* = 0.066), see Table 2. No additional patients developed a hypogammaglobulinemia during the five year follow-up (HR 1.95 (95% CI 0.86–4.45), *P* = 0.111). Indications for intravenous immunoglobulin (IVIG) treatment were a secondary immunodeficiency with severe hypogammaglobulinemia (IgG <4.0 g/L) or mild to moderate hypogammaglobulinemia (IgG 4.0–7.0 g/L) in combination with infections, or additional immunomodulating treatment for AAV. In the RTX-CYC group, thirteen (38%) patients received IVIG in the first two years after remission-induction vs. seven (25%) in the RTX-only group (*P* = 0.267). During the five year follow-up, a total of eighteen patients received IVIG in the RTX-CYC group and eight patients in the RTX only group (*P* = 0.093).

### 3.5 Renal outcome

The median delta estimated glomerular filtration rates (eGFR) were available in 20 (70%) patients in the RTX-CYC group and in 8 (47%) in the RTX only group during clinical remission after two years of follow-up. These levels did not differ between the two treatment groups (−1.0 [IQR, −19.0–7.0] ml/min/1.73 m^2^ vs. −0.5 [IQR, −13.5–10.0] ml/min/1.73 m^2^, *P* = 0.836).

One (3%) patient presented with ESRD at inclusion and was treated with RTX-CYC, but remained dialysis dependent. Within the first two years after remission-induction, one (3%) patient in the RTX-CYC group and one (4%) in the RTX only group developed ESRD (Table 2). One additional RTX-CYC treated patient developed ESRD after 36 months of follow-up (Table 3, Appendix Table B1).

### 3.6 Malignancy

One (3%) patient treated with RTX-CYC developed merkel cell carcinoma within one year after treatment. This patient was treated with cyclophosphamide for previous disease presentations. Two (7%) patients treated with RTX only developed malignancies within two years. Of those patients, one patients had de novo AAV and was diagnosed with prostate cancer a few months after remission-induction therapy. The other patient was previously treated with CYC and developed dermal basal cell carcinoma after two years. No additional malignancies were diagnosed during the five year follow-up (Table 2, Table 3).

### 3.7 Mortality

A total of six (10%) patients died during the two year follow-up, of which five (83%) were treated with RTX-CYC. Two more patients died during the five year follow-up, one was treated with RTX only and one with RTX-CYC (Fig. 3B). Mortality rate did not differ significanty between groups at the two and five year follow-up (Table 2, Table 3). Causes of death are depicted in Appendix Table C1.

## 4 Discussion

There is a need for a delicate balance between sufficient treatment and the prevention of adverse events in patients with AAV. Optimal treatment entails reaching remission, but also maintenance of remission by prevention of ongoing organ damage. The kidney is often affected and ongoing organ damage may have a major impact on the quality of life and mortality risk [[18], [19], [20], [21]]. We conducted a retrospective study to evaluate the long-term efficacy and safety of remission-induction with RTX only or with a combination of RTX and low dose CYC, both followed by tailored maintenance with RTX, in patients with AAV. Two years after remission-induction therapy, we observed significanty more relapses in patients treated with RTX only, with an equal number of patients receiving RTX maintenance in both groups at the same time after remission-induction. This difference disappeared after five years of follow-up. There was no significant difference in the occurrence of adverse events. Our findings implicate that addition of low dose CYC to RTX might have a beneficial effect on the prevention of major relapses, without increasing the risk of adverse events.

Previous studies comparing RTX with CYC, or a combination of RTX and CYC with CYC, as remission-induction therapy, showed comparable results in remission and total relapse rates within 18–24 months [4,5,12]. Randomized controlled trials that compare the efficacy and safety between RTX only and RTX with CYC have not yet been performed, but reported major relapse rates are similar to our study [4,5,11,12]. In the follow-up of the RAVE trial [11], 18% of the patients with renal involvement who were treated with RTX only and reached remission after six months, had a major relapse within 18 months, compared to 24% within 24 months in the current study. However, an additional 37% of the patients in the RAVE trial had a minor relapse, for which they received therapy. This might have lowered the risk of a major relapse. Another possible explanation is that a larger proportion of the patients in the current study had relapsing disease at time of inclusion (68% vs. 51%), which has been suggested to be a predictor of future relapse in previous studies [22]. During the two year follow-up in the RITUXVAS trial [12], 3% of the patients treated with a combination of RTX and CYC had a major relapse, which is consistent with 3% in the current study. Of note, all patients in the RTUXVAS trial were newly diagnosed with renal involvement and did not receive maintenance treatment.

Furthermore, in the current study, patients in the RTX-CYC group tended to have a more severe course of disease. This is reflected by a higher proportion of renal involvement in these patients. For this reason, seven patients in this treatment group received additional therapy with plasma exchange. Whether this could explain the lower relapse rate is questionable, since the clinical value of plasma exchange, especially in patients with chronic renal damage, was recently shown to be limited [23]. Although not statistically different, more patients died during the first two years in the RTX-CYC group than in the RTX only group. This could have affected our outcome, since these patients could potentially have had a disease relapse during longer follow-up. After five years, there is no difference in relapse rate between the two treatment groups. This means that patients treated with the combination RTX-CYC relapse after the first two years after remission-induction, which implicates that the effect of this combined treatment is longer-lasting than of RTX alone.

We did not find differences in adverse events, including hypogammaglobulinemia, infections, ESRD, and malignancy between patients treated with RTX-CYC or with RTX-only. IgG levels decreased equally in both groups and the proportion of patients with hypogammaglobulinemia did not differ significantly. However, there was a trend towards an additional effect of CYC on IgG levels, which is also reflected in the number of patients receiving IVIG suppletion. The stronger effect of the combination of RTX and CYC on IgG levels is supported by a study reporting that IgG levels are lower when RTX and CYC are combined as opposed to treatment with CYC alone [24]. Importantly, this was not associated with an increased risk of infections in our study and the observed infection rates were in concordance with previous studies [4,12]. It has been reported that serious infectious complications mainly present as respiratory tract infections in patients with AAV treated with RTX, which is consistent with results of the current study [25]. Immunosuppressive therapy does not only increase infection risk, but also decreases the ability of the immune system to adequately detect and kill malignant cells [26,27]. This raises concerns about cancer risk in patients treated with immunosuppressants. Particularly patients treated with a high cumulative dose of CYC have an increased risk for malignancies [26]. In general, the risk of developing malignancies in AAV patients is increased, but seems comparable across treatment groups, at least in the first years after remission-induction therapy [4,12,26].

Our study has several limitations to address that are mainly related to the sample size and the retrospective nature. First, the groups are too small to create a prediction model and to identify possible risk factors for relapse and other adverse events. Secondly, more patients in the RTX-CYC group had renal involvement, and clinicians tend to give CYC more frequently in patients with severe or rapidly progressive disease presentations [28]. However, despite the larger proportion of severe disease in this treatment group, relapse rates in the first two years were lower, which supports our findings that the addition of CYC to RTX is effective in prolonging relapse-free survival. A major strength of the current study is that it gives insight into treatment outcomes over a relatively long time-span, in which all patients were followed for at least two years. Furthermore, we only included patients who received tailored maintenance therapy with RTX to minimize heterogeneity between the two groups.

In conclusion, we compared long-term outcomes between AAV patients treated with RTX-CYC and patients treated with RTX only, both followed by a tailored maintenance therapy with RTX. Results implicate that adding low dose CYC to RTX might be beneficial in preventing disease relapse in patients with AAV in the short-term. The results may support clinical decision making and contribute to future research. Randomized controlled trials that compare the efficacy and safety between RTX and a combination of RTX with CYC are needed.

## CRediT author statement

Renée Ysermans: conceptualization, methodology, formal analysis, investigation, resources, writing – original draft, visualization. Matthias Busch: conceptualization, methodology, formal analysis, investigation, resources, writing – review and editing. Joop Aendekerk: investigation, resources, writing – review and editing.Jan Damoiseaux: methodology, supervision, writing – review and editing. Pieter van Paassen: conceptualization, methodology, resources, supervision, writing – review and editing.

## Declaration of competing interest

The authors declare that they have no known competing financial interests or personal relationships that could have appeared to influence the work reported in this paper.

## Data Availability

Data will be made available on request.
