# Supplementary material for: Adding low dose cyclophosphamide to rituximab for remission-induction may prolong relapse-free survival in patients with ANCA vasculitis: A retrospective study
Source: J Transl Autoimmun. 2022 Dec 15;6:100178. doi: 10.1016/j.jtauto.2022.100178 (PMC9800337; doi:10.1016/j.jtauto.2022.100178)
Supplement: Supplementary file 1 [file mmc1.docx]

**Appendix A

Table A.1.** Infection types during the five year follow-up.

| **Infection type** | **RTX-CYC (N = 12)** | **RTX only (N = 15)** |
| --- | --- | --- |
| **Respiratory tract infections** |  |  |
| Pseudomonas | 2 | - |
| CMV | - | - |
| Pneumocystis | - | 1 |
| Influenza | - | 1 |
| Unknown micro-organism | 5 | 4 |
| **Upper gastrointestinal-tract infections** |  |  |
| HSV stomatitis | 1 | - |
| Candida oesophagitis | - | 1 |
| **Lower gastrointestinal tract infections** |  |  |
| Blastocystis hominis | - | 1 |
| Clostridium difficile | 1 | 1 |
| Hepatitis E | - | 1 |
| Candida glabrata | 1 | - |
| Unknown micro-organism | - | 1 |
| **Urinary tract infections** |  |  |
| Escherichia coli | 1 | 1 |
| Candida albicans | - | 1 |
| **Skin infections** |  |  |
| Mycobacterium marinum | - | 1 |
| **Other^a^** |  |  |
| Keratitis | 1 | - |
| Otitis media | - | 1 |

Abbreviations: RTX = rituximab, CYC = cyclophosphamide.
*Micro-organism unknown.
