# Supplementary material for: Adding low dose cyclophosphamide to rituximab for remission-induction may prolong relapse-free survival in patients with ANCA vasculitis: A retrospective study
Source: J Transl Autoimmun. 2022 Dec 15;6:100178. doi: 10.1016/j.jtauto.2022.100178 (PMC9800337; doi:10.1016/j.jtauto.2022.100178)
Supplement: Supplementary file 2 [file mmc2.docx]

**Appendix B

Table B.1.** Patients with end stage renal disease.

| **Treatment** | **Baseline eGFR (**ml/min/1,73m^2^) | **eGFR during ESRD (**ml/min/1,73m^2^) | **Months after disease presentation** | **ANCA renal risk score** |
| --- | --- | --- | --- | --- |
| RTX-CYC | - | - | During disease presentation | NA |
| RTX-CYC | 12 | 8 | 11 | High |
| RTX-CYC | 15 | 8 | 36 | High |
| RTX only | 29 | 14 | 8 | Moderate |

Abbreviations: RTX = rituximab, CYC = cyclophosphamide, eGFR = estimated glomerular filtration rate, ESRD = end stage renal disease, ANCA = anti-neutrophil cytoplasmic antibody .
