# Supplementary material for: Adding low dose cyclophosphamide to rituximab for remission-induction may prolong relapse-free survival in patients with ANCA vasculitis: A retrospective study
Source: J Transl Autoimmun. 2022 Dec 15;6:100178. doi: 10.1016/j.jtauto.2022.100178 (PMC9800337; doi:10.1016/j.jtauto.2022.100178)
Supplement: Supplementary file 3 [file mmc3.docx]

**Appendix C

Table C.1.** Causes of death.

| **Age** | **Treatment** | **Months after remission-induction** | **Cause of death** |
| --- | --- | --- | --- |
| 71 | RTC-CYC | 1 | Multiple organ failure, cytomegalovirus pneumonia and AAV disease activity |
| 68 | RTC-CYC | 4 | Not known |
| 79 | RTX-CYC | 6 | AAV disease activity and pseudomonas pneumonia |
| 69 | RTC-CYC | 8 | Not known |
| 51 | RTX-CYC | 18 | Not known |
| 84 | RTX-CYC | 58 | Septic shock |
| 60 | RTX only | 3 | Haemorrhagic shock and pneumocystis jiroveci pneumonia |
| 72 | RTX only | 55 | Acute heart failure |

Abbreviations: RTX = rituximab, CYC = cyclophosphamide.
